# Profile of Maternal and Foetal Complications during Labour and Delivery among Women Giving Birth in Hospitals in Matlab and Chandpur, Bangladesh

**DOI:** 10.3329/jhpn.v30i2.11295

**Published:** 2012-06

**Authors:** Fauzia Akhter Huda, Anisuddin Ahmed, Sushil Kanta Dasgupta, Musharrat Jahan, Jannatul Ferdous, Marge Koblinsky, Carine Ronsmans, Mahbub Elahi Chowdhury

**Affiliations:** ^1^icddr,b, GPO Box 128, Dhaka 1000, Bangladesh; ^2^John Snow Inc., Arlington, Virginia, USA; ^3^London School of Hygiene & Tropical Medicine, London, UK

**Keywords:** Caesarean section, Delivery, Foetal complications, Hospitals, Maternal complications, Maternal mortality, Perinatal mortality, Record-keeping, Bangladesh

## Abstract

Worldwide, for an estimated 358,000 women, pregnancy and childbirth end in death and mourning, and beyond these maternal deaths, 9-10% of pregnant women or about 14 million women per year suffer from acute maternal complications. This paper documents the types and severity of maternal and foetal complications among women who gave birth in hospitals in Matlab and Chandpur, Bangladesh, during 2007-2008. The Community Health Research Workers (CHRWs) of the icddr,b service area in Matlab prospectively collected data for the study from 4,817 women on their places of delivery and pregnancy outcomes. Of them, 3,010 (62.5%) gave birth in different hospitals in Matlab and/or Chandpur and beyond. Review of hospital-records was attempted for 2,102 women who gave birth only in the Matlab Hospital of icddr,b and in other public and private hospitals in the Matlab and Chandpur area. Among those, 1,927 (91.7%) records were found and reviewed by a physician. By reviewing the hospital-records, 7.3% of the women (n=1,927) who gave birth in the local hospitals were diagnosed with a severe maternal complication, and 16.1% with a less-severe maternal complication. Abortion cases—either spontaneous or induced—were excluded from the analysis. Over 12% of all births were delivered by caesarean section (CS). For a substantial proportion (12.5%) of CS, no clear medical indication was recorded in the hospital-register. Twelve maternal deaths occurred during the study period; most (83%) of them had been in contact with a hospital before death. Recommendations include standardization of the hospital record-keeping system, proper monitoring of indications of CS, and introduction of maternal death audit for further improvement of the quality of care in public and private hospitals in rural Bangladesh.

## INTRODUCTION

Giving birth should be a time for celebration; however, for an estimated 358,000 women worldwide, pregnancy and childbirth end in death and mourning (1,2). Beyond these maternal deaths are numerous episodes of acute maternal complication: by some estimates, 9-10% of pregnant women or about 14 million women per year suffer from acute maternal complications (2,3). Estimate of the World Health Organization (WHO), United Nations Children's Fund, and United Nations Population Fund (approximately 15% of expected births suffering from obstetric complications) is more than double this figure: approximately 20 million women suffer from an obstetric complication. The consequences of birth and acute maternal complications, including death and disabilities, make up the largest burden of disease affecting women in developing countries (4-6).

In Bangladesh, an estimated 11,000-21,000 women die each year due to pregnancy-related complications (7), and a further 320,000 women suffer from injuries or disabilities caused by these complications during pregnancy and childbirth (8). Although most of these injuries or disabilities are not life-threatening, these may render women outcast from their family and society. Women with disabilities may also face cultural, social or other barriers to obtaining care and, therefore, become silent sufferers (9-11).

Measuring acute maternal complication is difficult, particularly in populations where not all women give birth in a hospital. The number and percentage of women in Bangladesh who suffer from acute maternal complications or medium or long-term disabilities are not yet known. The reliability of reported complications based on a woman's recall is poor, even if the woman suffered from a life-threatening complication (12,13). Reliable ascertainment of maternal complication requires observation by a trained service provider, and this is typically facility-based. For this reason, few studies have been able to measure the incidence of acute maternal complications at the population level (14,15).

The aim of the present study was to document the types and severity of acute maternal and foetal complications among women admitted to different hospitals around the time of childbirth and postpartum.

## MATERIALS AND METHODS

### Study area

We conducted the study in the icddr,b service area in Matlab, a rural area located about 55 km southeast of Dhaka, the capital of Bangladesh. In 2007, the population in the study area was approximately 113,660 (16). The major sources of income are fishing and farming, and about two-thirds of women have received institutional education (16). The area has been under surveillance since 1966 for vital events (births, deaths, marriages, migration) by the village-based Community Health Research Workers (CHRWs). At the time of this study, the CHRWs visited each household bi-monthly to collect data on the reproductive health status of women and determined pregnancy, using a pregnancy-detection strip. Other CHRWs provided services from fixed-site clinics bi-weekly, along with counselling pregnant women to seek antenatal care and attend hospital for safe delivery. These CHRWs also disseminated information to groups of pregnant women about home-based lifesaving skills for newborns, including management of the newborns during normal delivery and for maternal and neonatal complications, such as prolonged labour, excessive bleeding, and birth asphyxia (17-19).

The icddr,b service area has four subcentre clinics run by nurse-midwives and paramedical staff and a hospital in Matlab town with a 30-bed maternity unit run by doctors and nurses (16). Each subcentre clinic serves about 20,000 persons. They provide limited obstetric services 24 hours, including care for normal labour and delivery, the first dose of antibiotic for infection, the first dose of magnesium sulphate (MgSO_4_) for eclampsia, and oxytocin only for active management of third stage of labour (AMTSL). When necessary, they refer women with complications to the Matlab Hospital of icddr,b where the staff members provide all components of basic emergency obstetric care (EmOC), including manual removal of the placenta, assisted delivery, oxytocin for AMTSL, MgSO_4_, and sedatives for eclampsia, and removal of retained products.

Complicated cases not manageable at the Matlab Hospital are referred to the public and private hospitals in Chandpur district town where services can be reached in about 40 minutes by motorized transport and in about one hour by three-wheelers from Matlab; icddr,b offers free transportation to all the patients referred but management of patients in hospitals in Chandpur is not part of the responsibility of icddr,b.

### Study population

We targeted all pregnant women in the icddr,b service area in Matlab, who gave birth during 2007-2008.

### Definition of maternal complications

Information on maternal complications was collected for all women who were admitted during labour or up to 42 days postpartum to any of the following hospitals: the Matlab Hospital of icddr,b, one public hospital in Matlab, and two public and 26 private hospitals in Chandpur district.

We aimed at classifying women by the severity of maternal complications, using three groups: severe maternal complication, less-severe maternal complication, and vaginal delivery without any maternal complication. The definition of severe maternal complication was adapted from the definitions of near-miss and lifesaving surgery proposed in the literature (20). Women with maternal complications were classified by primary diagnosis only, giving precedence to haemorrhage, hypertensive disorders of pregnancy, infection, anaemia, and dystocia sequentially. Indications for CS were classified using the classification proposed by Stanton *et al.* (21). Some women had given birth by CS without a reported maternal complication; these were included in a category of CS without any maternal complication. Lastly, we added a category of foetal complication, regardless of whether or not women had a maternal complication (Table 1).

### Data-collection

As part of the regular responsibilities, the CHRWs generated a list of pregnant women, using the women's unique identifiers. They collected information within one or two week(s) of a pregnancy outcome on all pregnant women during 2007-2008, including the place of birth, and the name and location of the hospital if any admission took place during delivery or immediately postpartum. Admissions to more than one hospital were noted. They also noted whether the child was born alive and survived or not until the first week of life.

**Table 1.** Diagnostic criteria for maternal and foetal complications

**Severe maternal complication:**	
Dystocia	Major obstetric interventions (emergency and elective CS, laparotomy, hysterectomy, craniotomy, and internal version) due to absolute maternal indications (Ruptured uterus; Brow presentation; Transverse lie; and Foetopelvic disproportion, including impending rupture of uterus) (22)
Haemorrhage	Severe antepartum and postpartum haemorrhage (bleeding with shock or transfused with 2 or more units of blood)
Hypertensive disorders of pregnancy	Eclampsia (seizures associated with hypertension, i.e. diastolic blood pressure ≥110 mmHg) and severe pre-eclampsia (hypertension with proteinuria >2 ++, blurred vision, or hyperreflexia)
Septic shock or septicaemia	Genital source of infection and hyperthermia (fever 38.3 °C and above for >48 hours) or hypothermia and low blood pressure (systolic <90 mmHg) or confusion or unconsciousness or scanty urine output (<30 mL/hour) and faster pulse rate (110 or more/minute) or rapid breathing (30 or more breaths/minute)
Severe anaemia	Haemoglobin level <7 g/dL
**Less-severe maternal complication:**	
Women who did not meet the criteria for severe maternal complications but hospital-records reported a diagnosis of vaginal bleeding, dystocia (prolonged labour, breech delivery, and other malpresentations which do not qualify as being due to absolute maternal indications), hypertension, infection (chorioamnionitis and urinary tract or genital infection), or mild or moderate anaemia	
**Vaginal delivery without any maternal complication:**	
Women who did not meet the criteria for severe or less-severe maternal complications and gave birth vaginally	
**CS without any maternal complication:**	
CS without any of the maternal complications mentioned above	
**Foetal complication:**	
Perinatal death (stillbirth* or death within the first week of life), foetal distress†, cord prolapse$, twin delivery, and premature delivery§ whether or not there was a maternal complication	

*Birth of a foetus after 28th completed weeks (weighing 1,000 g or more) when the baby does not breathe or show any sign of life after delivery

†A clinical condition characterized by a complex of signs indicating a critical response in the foetus to stress, a result of intrauterine foetal hypoxia

$The umbilical cord lying inside the vagina or outside the vulva following rupture of the membrane

§Delivery of the foetus before 36th completed weeks of pregnancy (23-25); CS=Caesarean section

A physician searched the hospital-records for any Matlab woman admitted during labour or postpartum to the Matlab Hospital, or any of the public or private hospitals in Matlab or Chandpur, including the admission registers and individual patient-records. Admissions were classified by maternal or foetal complications (Table 1). The hospital-records were reviewed every two weeks, using a structured data-extraction form.

Maternal deaths were noted by the CHRWs and matched with maternal death reporting from the Matlab's Health and Demographic Surveillance System (HDSS). The cause of death was ascertained by verbal autopsy diagnosed by a physician (26). Data on maternal education were also obtained from the HDSS.

### Analysis of data

Data were computerized using the Oracle 10g software (Oracle Corporation, USA) after review for accuracy, consistency, and completeness. Descriptive analyses of types of complications and care-seeking patterns were done using the Stata software (version 8.0) (Stata Corp. Inc., College Station, TX, USA). Pearson's chi-square test was used for testing the crude associations.

## RESULTS

### Description of the sample

**Fig. 1.**
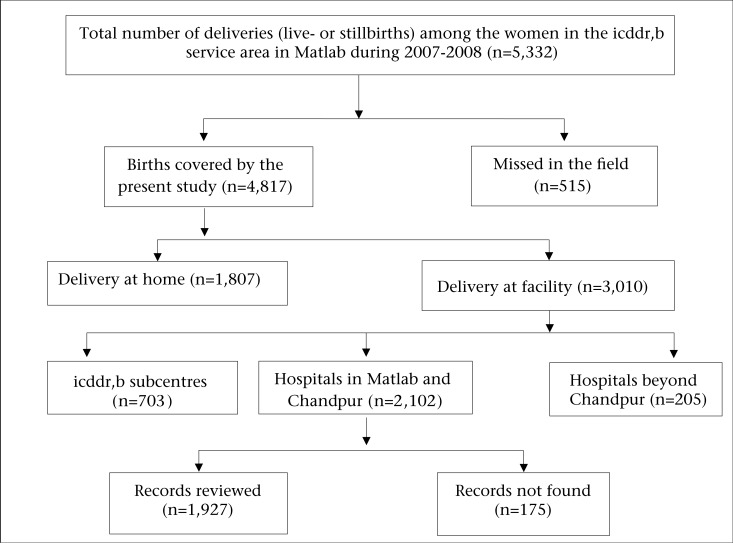
Study coverage of women in the icddr,b service area in Matlab, who delivered during 2007-2008

In the study area, the CHRWs reported 5,332 women with a pregnancy outcome during 2007-2008, of whom 4,817 (90%) were covered by the present study (Fig. 1). Of these women, 3,010 (62.5%) delivered in facilities and 1,807 (37.5%) delivered at home.

Review of the facility-records was not feasible to perform for all women who delivered in different facilities (n=3,010). Records were reviewed for the 2,102 women who gave birth in the local hospitals, including the Matlab Hospital of icddr,b, one public hospital in Matlab, and two public and 26 private hospitals in Chandpur. The remaining 908 women who delivered at the icddr,b subcentre clinics (n=703) and at hospitals beyond Chandpur (n=205) were excluded from this record-review procedure primarily because: (a) complicated deliveries get referred from the subcentres to the Matlab Hospital of icddr,b; (b) women who sought care at hospitals beyond Chandpur included more than 98 different hospitals across the country, including districts as far as Dhaka, Cox's Bazar, Sylhet, Khulna, and Dinajpur—the project did not have the funds to track them; and (c) in some cases, women themselves did not know or remember the name of the hospital. Finally, among the 2,102 women who delivered in the local hospitals, 1,927 (91.7%) hospital-records were reviewed, and 175 (8.3%) records were not found.

### Hospital-delivery and referral pattern

**Fig. 2.**
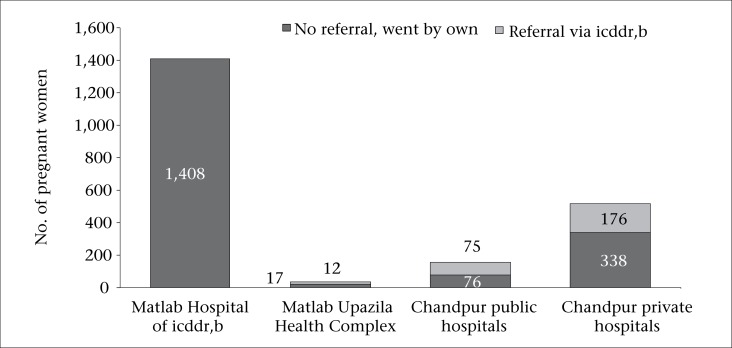
Place of delivery and referral pattern in women giving birth in Matlab and Chandpur hospitals, 2007-2008

Of the 2,102 women, 1,408 (67%) attended the Matlab Hospital of icddr,b and the remaining 694 (33%) women gave birth in the public or private hospitals in Matlab and Chandpur. More than one-third (38%) in the latter group were referred from the Matlab Hospital of icddr,b; the remaining 62% bypassed the icddr,b health system (Fig. 2). The use of public hospitals was low [29/2,102 in Matlab Upazila Health Complex (UHC) and 151/2,102 in Chandpur public hospitals]; 514 of the 2,102 births took place in the local hospital in the private sector.

### Maternal complications

Table 2 reveals the profile of women with maternal complications and the places of care-seeking for such complications. Women with severe (75.2%) and less-severe (49.5%) maternal complications sought care in the private hospitals in Chandpur. Women who had a vaginal delivery without any maternal complication were mostly (91%) managed in the Matlab Hospital of icddr,b.

**Table 2.** Women with maternal complications by severity and type of hospital in Matlab and Chandpur, 2007-2008

Maternal complication	Place of birth							
	Matlab Hospital of icddr,b		Public hospitals in Matlab and Chandpur		Private hospitals in Chandpur		All	
	No.	%	No.	%	No.	%	No.	%
Severe maternal complications	19	13.5	16	11.3	106	75.2	141	100
Less-severe maternal complications	102	32.8	55	17.7	154	49.5	311	100
CS without any maternal complication	0	0	13	8.7	137	91.3	150	100
Vaginal delivery without any maternal complication	1,201	90.6	67	5.1	57	4.3	1,325	100
No records found	86	49.1	29	16.6	60	34.3	175	100
All	1,408	67.0	180	8.6	514	24.5	2,102	100

CS=Caesarean section

The majority (68.8%) of severe and less-severe maternal complications was related to dystocia, and the bulk of those visited the private hospitals (Table 3). Hypertensive disorders were present in 12.6% of the women, and haemorrhage (8.4%), anaemia (6.4%), and infection (3.8%) were infrequent. The Matlab Hospital of icddr,b took the major share in treating women with any complication other than dystocia (Table 3). Very few cases of haemorrhage, hypertensive diseases, infection, or anaemia were treated in any of the public or private hospitals in Matlab or Chandpur.

**Table 3.** Severe and less-severe maternal complications by obstetric diagnosis and type of hospital in Matlab and Chandpur, 2007-2008

Maternal complication	Place of birth			
	Matlab Hospital of icddr,b (n=121)	Public hospitals in Matlab and Chandpur (n=71)	Private hospitals in Chandpur (n=260)	All (n=452)
Haemorrhage	17 (14.0)	7 (9.9)	14 (5.4)	38 (8.4)
Severe	6	1	11	18
Less severe	11	6	3	20
Hypertensive disorders of pregnancy	36 (29.7)	11 (15.5)	10 (3.9)	57 (12.6)
Severe	5	3	8	16
Less severe	31	8	2	41
Infection	13 (10.7)	2 (2.8)	2 (0.8)	17 (3.8)
Severe (sepsis)	0	0	1	1
Less severe	13	2	1	16
Anaemia	26 (21.5)	1 (1.4)	2 (0.8)	29 (6.4)
Severe	8	1	2	11
Less severe	18	0	0	18
Dystocia	29 (24.0)	50 (70.4)	232 (89.2)	311 (68.8)
Severe	0	11	84	95
Less severe	29	39	148	216
All	121 (100)	71 (100)	260 (100)	452 (100)

Figures in parentheses indicate percentages

### Relationship between maternal and foetal complications

We found 105 perinatal deaths among women giving birth in the hospitals, resulting in a hospital perinatal mortality rate of 5.0%. The perinatal mortality rate was the highest (7.1%) among women with severe maternal complications (Table 4). The rates of foetal distress were the highest among women with a CS without any maternal complication (32.7%), followed by women with severe and less-severe maternal complications (17% in severe and 13.8% in less severe).

### Indications for caesarean section

In total, 591 (12.3%) CS were recorded among all women in Matlab, of which 401 (68%) were carried out in the hospitals in Matlab or Chandpur. The remaining 190 (32%) CS were performed in hospitals beyond Chandpur, the indications for which remain unknown. The private hospitals in Chandpur performed nine times as many CS compared to the public hospitals in Matlab and Chandpur (Table 5). CS without any maternal complication was found in 24.9% of the cases. The next common indication for CS was an absolute maternal indication (24.7%), followed by other less-severe maternal complications (18%) and failure to progress (16.5%). For 12.5% of the CS, no clear medical indication was noted in the hospital-records (Table 5).

### Educational status of admitted women

The proportion of women with severe or less-severe maternal complications was strongly associated with the level of education of the women (Table 6). CS without any maternal complication was more common among women with more than 10 years of schooling.

### Maternal death

**Fig. 3.**
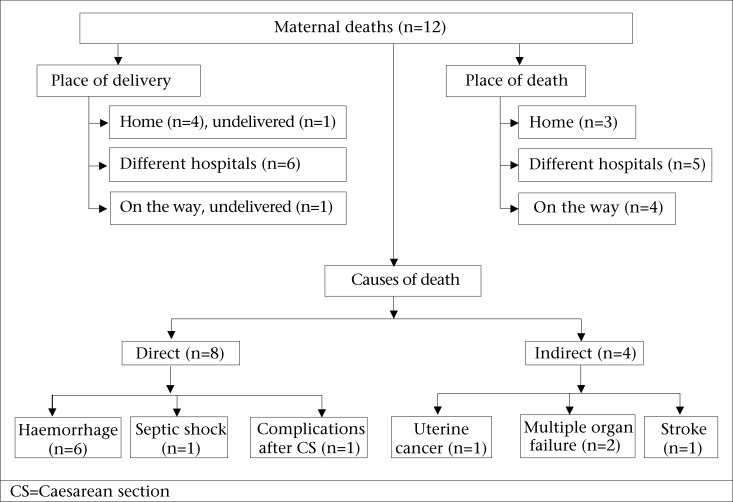
Maternal deaths in the icddr,b service area in Matlab, 2007-2008

Twelve maternal deaths were recorded in the study population during the two-year study period, resulting in a maternal mortality ratio of 255 per 100,000 livebirths. Four of the women who died delivered at home, and of them, three visited a hospital during the postpartum period. Six of the women delivered in a hospital, and two women died without delivery: one at home and one on the way to a hospital. Only one maternal death was found in the records of a private hospital, and this hospital-record was reviewed. Results of review of verbal autopsy data on all 12 deaths showed that six women died due to haemorrhage (one for antepartum haemorrhage and five for postpartum haemorrhage); one woman died from septic shock; one woman died from post-operative complication after CS; and four women died due to indirect causes (two from multiple organ failure and one each from uterine cancer and stroke) (Fig. 3).

**Table 4.** Maternal and foetal complications by severity in Matlab and Chandpur, 2007-2008

Maternal complication	Foetal complication						
	Perinatal death	Foetal distress	Cord prolapse	Twin pregnancy	Premature delivery	No foetal complications	All
Severe maternal complications	10 (7.1)	24 (17.0)	0	1 (0.7)	1 (0.7)	105 (74.5)	141 (100)
Less-severe maternal complications	12 (3.9)	43 (13.8)	8 (2.6)	5 (1.6)	5 (1.6)	238 (76.5)	311 (100)
CS without any maternal complication	7 (4.7)	49 (32.7)	0	2 (1.3)	0 (0)	92 (61.3)	150 (100)
Vaginal delivery without any maternal complication	59 (4.5)	22 (1.7)	110 (8.3)	6 (0.5)	17 (1.3)	1,111 (83.9)	1,325 (100)
No records found	17 (9.7)	0	0	0	0	158 (89.7)	175 (100)
All	105 (5.0)	138 (6.6)	118 (5.6)	14 (0.7)	23 (1.1)	1,704 (81.0)	2,102 (100)

Figures in parentheses indicate percentages; CS=Caesarean section

**Table 5.** Indications of CS in public and private hospitals in Matlab and Chandpur, 2007-2008

Indications of CS	Place of birth		
	Public hospitals in Matlab and Chandpur (%)	Private hospitals in Chandpur (%)	All (%)
Absolute maternal indication*	27.5	24.7	24.7
Other severe maternal complications†	5.0	3.0	3.2
Other less-severe maternal complication$	10.0	18.8	18.0
CS without any maternal complications§	12.5	26.3	24.9
Failure to progress	25.0	15.5	16.5
No clear medical indication**	20.0	11.6	12.5
All	40 (100)	361 (100)	401 (100)

*Ruptured uterus resulting in haemorrhage and shock, brow presentation, transverse lie, foetopelvic disproportion, including pre-rupture of uterus

†Include eclampsia and severe pre-eclampsia

$Include prolonged labour, history of previous CS, breech presentation, and other malpresentations which do not qualify as being due to absolute maternal indications, hypertension

§Include foetal distress, premature rupture of the membrane, disorders of amniotic fluid, and membrane

**Include post-dated, premature labour, and full-term pregnancy with labour pain; CS=Caesarean section

## DISCUSSION

Nearly two-thirds (62.5%) of all pregnant women in Matlab of Bangladesh gave birth in hospital. Review of the hospital-records showed that 7.3% of the admitted women were diagnosed with a severe maternal complication while 16.1% had a less-severe maternal complication. The great majority of severe and less-severe maternal complications were related to dystocia (68.8%), followed by hypertensive disorders of pregnancy (12.6%); haemorrhage, anaemia, and infection were less common. If these hospital cases represent the lower bound of the number of women with severe and less-severe maternal complications in the population, at least 2.9% of all pregnant women suffer from a severe maternal complication and 6.5% a less severe maternal complication (excluding those who were diagnosed with an abortion-related complication). Women with severe maternal complications were also at a greater risk of perinatal mortality; hence, the burden of severe maternal morbidity is compounded by the concomitant burden of perinatal mortality. The higher perinatal mortality among women with maternal complication confirms findings elsewhere (27).

**Table 6.** Maternal complications by maternal education in Matlab and Chandpur, 2007-2008

Maternal complication	Years of schooling											
	No schooling		1-4		5-7		8-10		>10		Unknown	
	No.	%	No.	%	No.	%	No.	%	No.	%	No.	%
Severe maternal complications	11	5.3	16	10.3	31	6.4	54	6.1	12	8.8	17	7.3
Less-severe maternal complications	37	17.9	14	9.0	60	12.4	147	16.5	24	17.7	29	12.4
CS without any maternal complication	13	6.3	10	6.5	19	3.9	70	7.9	22	16.2	16	6.9
Vaginal delivery without any maternal complication	136	65.7	103	66.4	342	70.9	543	61.1	64	47.1	137	58.8
No records found	10	4.8	12	7.7	30	6.2	75	8.4	14	10.3	34	14.6
All	207	100	155	100	482	100	889	100	136	100	233	100

p<0.01; CS=Caesarean section

Twelve maternal deaths occurred during the study period: for every maternal death, there were 12 women with severe maternal complications and 26 with less-severe maternal complications. If these ratios hold true worldwide, taking into account the WHO estimate of 358,000 maternal deaths annually (1,2), there would be 4.3 million women with severe maternal complications and 9.3 million with less-severe maternal complications (excluding abortion-related complications). It was found that haemorrhage caused 50% of maternal deaths, most (83%) of whom sought care from a hospital around birth or in the postpartum period. The findings of the present study showed that, despite the higher numbers of women presenting at hospitals with dystocia and hypertensive disorders of pregnancy, it continues to be haemorrhage that kills pregnant women in the Matlab setting. The common belief that complications during pregnancy are caused by supernatural factors that cannot be treated by medical doctors (28) appears to be changing: 75% of women who died had reached at least one hospital.

The findings of the present study showed that the less-severe maternal complications were not associated with excess perinatal mortality. This is surprising given that 48% of the study women with less-severe maternal complications were associated with dystocia, which is known to compromise the condition of the foetus. Even so, it is worth noting that most perinatal deaths occurred among women who suffered no complication and had a vaginal delivery—the findings being similar to those of other studies (27). This confirms that obstetric complications only explain part of all perinatal deaths.

Institutional delivery and/or the presence of a skilled birth attendant can make a critical difference to the survival of mothers and their babies as has been noted in other studies (29). The aim of future health programmes should be directed to sensitize people for delivery at facility with the provision of skilled attendance at birth. Given the 20-year history of a midwifery programme in the icddr,b service area in Matlab, it is encouraging that 62.5% of all pregnant women in the icddr,b service area gave birth in a facility compared to the 2007 nationwide rate of 15% (range 11-27%) (30). Of the total deliveries, the Matlab Hospital of icddr,b and its four subcentre clinics covered 44% of the total deliveries; 26 private hospitals in Chandpur covered another 10.6%; three public hospitals in Matlab and Chandpur covered only 3.7%; and the hospitals beyond Chandpur covered 4.2%. If the Matlab Hospital of icddr,b and its four subcentre clinics were removed from the equation, the institutional delivery rate of 18.5% in the other hospitals in Matlab and Chandpur and beyond is almost similar to that reported for the nation. Findings of studies in South Asian countries showed that it is only in Sri Lanka that most births take place in institutions compared to 21% in Nepal and less than 40% in India and Pakistan (31).

Women with a complication tend to bypass the icddr,b health system in Matlab, although the icddr,b system can identify, stabilize, and refer them to Chandpur (primarily to the public hospital) with transport paid. This bypassing phenomenon noted in some other studies needs further investigation (32). It is notable that most births taking place in the Matlab Hospital of icddr,b are normal vaginal deliveries while those with complications seek care in hospitals with comprehensive EmOC. It is likely that women and their families detect problems at home and determine to go directly to a hospital where they know they can have all emergency care, if needed.

Among all Matlab women with live- and stillbirths, 12% had a CS compared to the national rate of 7% (range 4-10%) (30). Those who sought care in the private hospitals were far more likely to have a CS than those in the public hospitals. This resulted in nine CS in the private hospitals for every one in the public hospitals. The share of CS in the public and private hospitals is similar to that found countrywide: about two-thirds of all CS were done in the private sector in 2007 (31)—nearly twice the rate found in the 2001-2003 national survey (33). Most women with dystocia and with other severe and less-severe maternal complications or foetal complications (e.g. foetal distress, breech presentation) visited a private hospital, and the majority of them had a CS. The diagnosis and treatment of dystocia is obviously an important contributor to the CS level. One study has shown that changes in diagnosing and managing dystocia cases could reduce the CS rate by 50% (34).

Another possible explanation for the high level of CS is the provider's incentive to pursue surgical delivery as identified by other studies (8,35). A number of CS with no clear medical indication (12.5% of cases) were identified by the present study. Obstetricians (and the private hospitals where they work) earn more money by doing CS than conducting vaginal deliveries. Similar findings were reported in studies in Jordan, Egypt, and Brazil where data demonstrate that medical emergency is not always the primary reason for this expensive surgery (36-38).

CS may also reflect women's desire to avoid the arduous process of labour and delivery, or consider it modern, particularly when they are from affluent society and have a higher level of education. Women with 8-10 years of schooling in the present study were far more likely to undergo CS than women with no education—consistent with this hypothesis. Similar findings have been reported in other studies where the CS rate was higher among the least poor, well-educated and urban women (8).

### Limitations

This study had a number of limitations. During 2007-2008, the study covered only 90% of the total births in the study area. Triangulation with the Matlab surveillance data (HDSS data) revealed that the great majority of the cases were missed during the rainy season when most part of the study area was under water, and communication was particularly difficult. Furthermore, 8.3% of the hospital-records could not be found, and 4.2% of the women delivering in the hospitals beyond Chandpur also could not be traced. The latter is of concern since they may have represented women with severe maternal complications. The estimate for acute maternal complications reported in this study is, thus, conservative. Minimal data were available on abortion-related complications, and these were, thus, excluded from the analysis, despite their importance to maternal health.

The definitions of severe and less-severe maternal complications used in the study were adapted from the existing definitions of near-miss and access to lifesaving surgery. The standard guideline of the WHO for the definition of near-miss was not available at the time of the study (39), and hence, it was not followed here. Possible misclassification of severe and less-severe maternal complications due to poor-quality record-keeping in both public and private hospitals also cannot be excluded.

The study does not account for outcomes of home-deliveries or deliveries in the Matlab subcentre clinics, although such outcomes are obviously extremely important. Given the lack of record-keeping of home-births by trained professionals and paucity of information recorded in subcentre clinic registers, there was no way to accurately describe the risks of the study population. It is also anticipated that women with severe or less-severe complications would be referred to the Matlab Hospital of icddr,b.

### Conclusions

The results of the present study highlight areas for capacity-building in the healthcare system of Bangladesh, including record-keeping and longitudinal tracking of patient information across hospitals. Yet, even with the poor record-keeping, we know that women suffer most from complications of dystocia, a relatively new and surprising finding. However, it continues to be haemorrhage that kills women. The population level with severe and less-severe complications is at least 10%. Perinatal mortality is linked to severe maternal complications but also was found high among women with no recorded complications.

Most women who seek hospital care are selecting the private over public-sector hospitals, especially when they have complications. With the increase in the number of maternity patients, the rate of CS in the private sector is increasing alarmingly, the causes of which raise a number of questions about need vs financial incentives. And those with complications are bypassing the well-established icddr,b midwifery and hospital in Matlab when a complication arises.

Studies similar to the present one are necessary to understand where exactly the problems lie in the health systems and where the limitations are to save mothers and newborns.

### Recommendations

Deliveries at facility are strongly encouraged to ensure good outcomes for both mother and newborn at delivery and beyond. After 15 years of introduction of a strategy for delivery at facility, about one-third of women in Matlab continue to deliver at home. A large proportion of women, particularly with complications, bypass the safe motherhood programme of icddr,b, the reason for which is not clearly understood. These two problems should be investigated for further strengthening of the safe motherhood programme of the Matlab Hospital of icddr,b.

The majority of the maternal deaths took place at hospitals, the reasons behind this could include poor quality of care or women arriving late at the hospitals. Maternal death audits need to be introduced in both public and private hospitals for the improvement of the quality of maternal health services. Special emphasis on awareness-raising efforts at the community level about specific danger signs and sites of EmOC services must continue, and efforts need to be initiated to improve appropriate referral and coordination with appropriate hospitals.

The rising CS rate is another emerging concern. We recommend introducing monitoring of the indications of CS in both public and private hospitals to avoid unnecessary surgical interference. The reasons behind the failure of the public sector to serve women during emergency complications and the increased use of the private sector need to be addressed.

A substantial proportion of the hospital-records were missing which is also an indication of poor management of the health systems. We recommend introducing a uniform standardized record-keeping system in both public and private hospitals and regular use of data of the management information system to monitor the quality of services.

Considerations of the above recommendations will help improve not only the safe motherhood programme in Matlab but also overall maternal health services in hospitals in Bangladesh.

## ACKNOWLEDGEMENTS

The study was funded by the United States Agency for International Development (USAID) (Grant No. GHS-A00-0300019-00). The research team acknowledges with gratitude the commitment of USAID to icddr,b's research efforts. Dr. Mahbub Elahi Chowdhury was partially supported by the National Institutes of Health Fogarty International Center (Grant No. TW007587-04).

The project team is grateful to the valued members of all the hospitals of Matlab and Chandpur, and the Matlab HDSS of icddr,b for their generous support and cooperation. The team members would like to express their gratitude to Mr. Ahsan Kabir for his outstanding care and precision he demonstrated in coordinating the data-collection and field site management and to each of the field staff members for their competent collection of data. Ms Evelyn Ford deserves special mention for her assistance during writing this paper; her efforts are acknowledged with profound appreciation.
